# Study of the Electrical Behavior of CsPbBr_3_ Single Crystal and Films under Visible and High-Energy Photons

**DOI:** 10.1021/acsaom.4c00455

**Published:** 2025-03-20

**Authors:** Tahira Khan, Manas R. Gartia, Jianwei Wang, Jyotsna Sharma

**Affiliations:** † Department of Petroleum Engineering, Louisiana State University, Baton Rouge, Louisiana 70803, United States; ‡ Department of Mechanical and Industrial Engineering, Louisiana State University, Baton Rouge, Louisiana 70803, United States; § Department of Geology and Geophysics, Louisiana State University, Baton Rouge, Louisiana 70803, United States; ∥ Center for Computation and Technology, Louisiana State University, Baton Rouge, Louisiana 70803, United States

**Keywords:** CsPbBr_3_, single crystal, PMMA-doped CsPbBr_3_ film, crystal structure, electrical behavior, γ-radiation

## Abstract

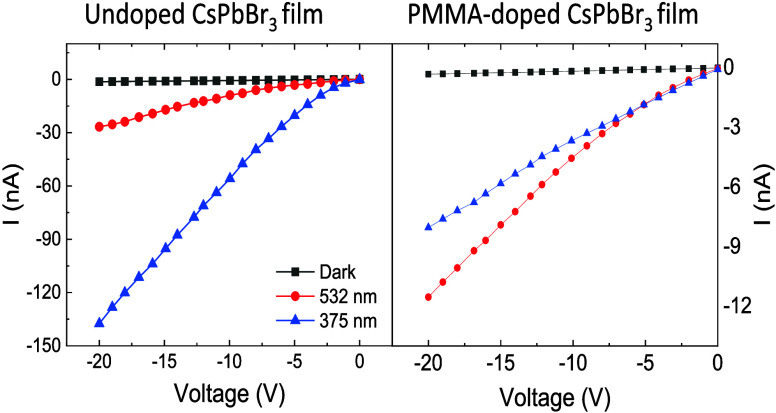

CsPbBr_3_ is a promising material due to its capability to detect high-energy radiation and applications in solar materials. A detailed study of the electrical behavior under γ-radiation is crucial for understanding the effects of radiation. In this work, we have studied the electrical behavior of CsPbBr_3_ single crystal and undoped and poly(methyl methacrylate) (PMMA)-doped films of CsPbBr_3_. We have introduced a new method for the growth of undoped and doped films. The X-ray diffraction (XRD) and energy-dispersive X-ray spectroscopy (EDS) analysis show the quality of the undoped, and PMMA-doped films is comparable to that of a single crystal (SC) based on purity. The current–voltage characteristic indicates that the SC and undoped film are more sensitive to ultraviolet light, but the PMMA-doped film is more sensitive to 532 nm. Also, the current under γ-radiation is lower than the dark current for SC and undoped film while is greater for PMMA-doped film when traced from 0 to −20 V. While the current–time characteristics indicate the current under γ-radiation is less negative than the dark current collected at −20 V for SC, undoped, and PMMA-doped films. The mobility-lifetime product is highest for SC, moderate for undoped film, and lowest for PMMA-doped film. These findings clarify some of the understanding of the device physics under visible and high-energy photons for optoelectronic and high-energy radiation detection.

## Introduction

CsPbBr_3_ is an inorganic metal halide compound which is widely studied due to its applications in solar cells and γ-radiation detection.^1−10^ CsPbBr_3_ is a high-temperature cubic material that undergoes a phase transition to a monoclinic structure at 136 °C (410 K) and a phase transition to another monoclinic structure at 24.85 °C (298 K).^11^ Among organic and inorganic metal halide-based perovskite materials, CsPbBr_3_ shows remarkable capability toward γ-ray detection, due to its high carrier mobility, large carrier diffusion lengths, and higher stability at high temperatures than organic perovskite materials.^1,8−10^ CsPbBr_3_ is a semiconductor material that works on the principle of electron–hole pair generation under γ-radiation and high-energy photons. So, studying the electrical behavior of the material is crucial for developing devices for specific applications under various environmental conditions and radiation. CsPbBr_3_ thin films are extensively studied due to their exceptional optoelectronic properties and stability, making them highly suitable for applications in devices such as solar cells, light-emitting diodes (LEDs), and photodetectors.^12−17^ With a direct bandgap of approximately 2.3 eV, CsPbBr_3_ efficiently absorbs visible light and generates charge carriers, contributing to high device efficiency. Compared with hybrid perovskites, all-inorganic CsPbBr_3_ offers enhanced thermal and environmental stability, allowing for better performance and longevity under real-world conditions. The thin-film structure also enables tunability through doping or nanostructuring, providing flexibility in optimizing their electrical and optical properties for specific applications. Additionally, their responsiveness to various photon types, including visible, ultraviolet, and γ-rays, makes CsPbBr_3_ thin films promising candidates for radiation detection and sensing technologies, further broadening their scope of use.^18−21^

There are many reports on the fabrication of the CsPbBr_3_ thin films from chemical methods, vapor deposition, and pulse laser deposition.^16,18−23^ The main drawback in chemical methods is obtaining a film that is free from impurities. CsPbBr_3_ is soluble only in dimethyl sulfoxide (DMSO) when the film is grown and annealed at low temperatures, resulting in films that contain the CsPbBr_3_ phase along with unwanted phases such as Cs_2_PbBr_4_ and PbBr_2_·2[(CH_3_)2SO].^24^ The other challenge is that the material has a salt-like nature and is sensitive to moisture and oxygen exposure, which makes it hard to get a good quality film and to use in different applications.^5,25,26^ Doping with poly(methyl methacrylate) (PMMA) to CsPbBr_3_ thin films introduces several beneficial changes in their properties. Primarily, PMMA acts as a protective layer, enhancing the film’s environmental stability by shielding it from moisture and oxygen and improving its resistance to thermal degradation.^27−29^ Additionally, PMMA can modify the optical properties, reducing recombination by passivating defects, which enhances photoluminescence and improves charge carrier dynamics.^27^ It also increases the film’s mechanical flexibility and durability, making it more suitable for flexible electronic applications.^19−21^ Several studies reported on PMMA-doped CsPbBr_3_ in terms of stability and optical spectroscopy.^27−30^ However, the literature lacks reports on a direct study of the changes in the electrical behavior of PMMA-doped CsPbBr_3_ which is one of the focuses of this study.

The interaction of visible, UV, and γ-radiation with materials can lead to various changes in their structural and electrical properties, depending on the energy of the photons involved. Visible light, being lower in energy, typically excites electrons in the material, leading to the generation of charge carriers, which is crucial for applications like solar cells and photodetectors.^31^ UV light, with higher energy, can cause stronger excitations and potentially induce defects or surface degradation, especially in sensitive materials, but it also plays an important role in UV-sensitive photodetectors. γ-radiation, being highly energetic, interacts deeply with the material, potentially displacing atoms and creating defects that significantly alter electrical conductivity, which is critical for radiation sensing technologies.^32,33^ In terms of electrical response, photon exposure can trigger various mechanisms such as charge carrier generation, defect creation, and recombination processes, which directly affect the conductivity and overall performance of materials like CsPbBr_3_.^33,34^ Previous studies on single crystal (SC) and films have shown that visible and UV photons enhance conductivity through photoinduced charge generation, while γ-photons often degrade material performance by introducing defects that trap charge carriers.^26,33,34^ Understanding these photonic interactions is essential for optimizing materials such as CsPbBr_3_ for applications in optoelectronics and radiation sensors.

There is extensive literature on photodetection and X-ray detection on thin films and the γ-radiation detection of a SC of CsPbBr_3_ by studying its γ radiation spectroscopy and the effects of different types of electrodes.^2,16,31,35−41^ The γ-radiation-induced hardness effects have also been studied in CsPbBr_3_ by using its electrical responses.^34^ This method involved the study of visible light on CsPbBr_3_ before and after exposure to a certain dose of γ-radiation. However, there are no studies that show directly how γ-radiation interacts and changes the electrical behavior of CsPbBr_3_ SC and films. Unfortunately, there is no direct study of the electrical response of γ-radiations in terms of *I*–*V* and *I*–*t* characteristics of CsPbBr_3_ SC and thin films. This kind of study, a focus of this study, provides direct information about the electrical behavior of material when exposed to γ-radiations and defects and traps.

In this study, we report a different approach for synthesizing CsPbBr_3_ undoped and PMMA-doped films from chemical methods. This study uses a multitechnique approach involving X-ray diffraction (XRD), energy-dispersive X-ray spectroscopy (EDS), thermogravimetric analysis (TGA), and differential scanning calorimetry (DSC) to correlate the structural, compositional, and thermal properties of thin films with their electrical responses under radiation. This study aims to investigate and compare the electrical behavior of CsPbBr_3_ SC, undoped CsPbBr_3_ films, and PMMA-doped CsPbBr_3_ films when exposed to visible, UV, and γ-photons. The primary motivation is to help us understand how photon type and material doping influence the charge carrier dynamics, conductivity, and overall electrical performance of CsPbBr_3_ SC, undoped CsPbBr_3_ films, and PMMA-doped CsPbBr_3_ films. These findings offer fundamental insights into the electrical response of the material, which could aid the development of radiation sensors, electronic devices for extreme environments, and materials for nuclear safety applications.

## Methods

### Single Crystal and Thin Film Preparation

**Figure 1:**
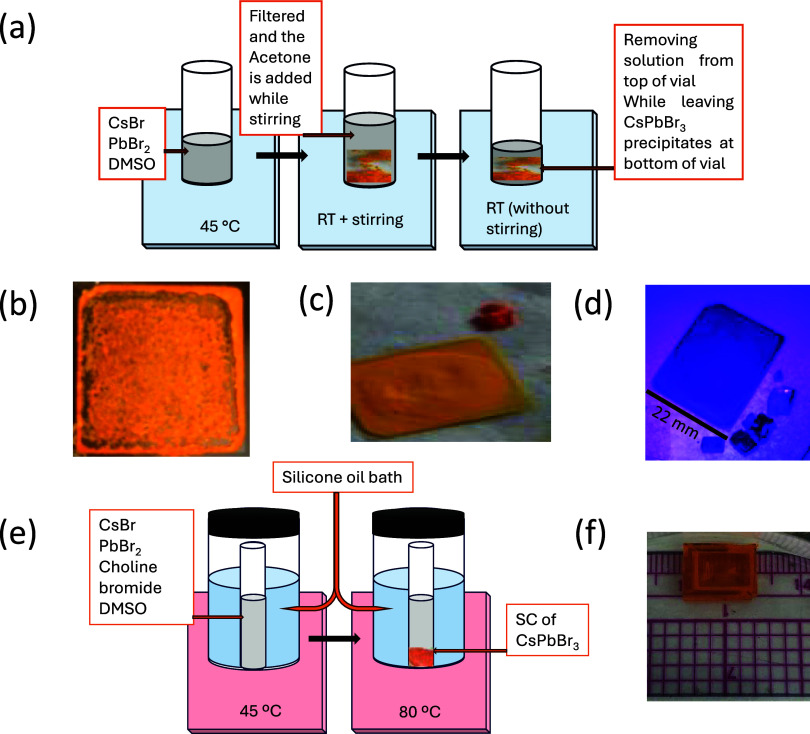
(a) Schematics to get CsPbBr_3_ precipitates for fabrication of films. (b) Optical image of the film obtained by the drip and dried method. (c) Optical image of the PMMA-doped CsPbBr_3_ film and a SC under visible and (d) UV light. Both SC and PMMA-doped films produce a purple color, showing that the quality of the CsPbBr_3_ film is comparable to the SCs. (e) Schematics to get CsPbBr_3_ SC from the ITC method at 80 °C. (f) Optical image of CsPbBr_3_ SC.

The procedure for the formation of films was performed in two steps. In the first step, the precipitates of CsPbBr_3_ were prepared by the method shown in Figure 1a. Cesium bromide (CsBr) and lead bromide (PbBr_2_) were procured from Sigma-Aldrich and used without further purification. A precursor solution of CsBr and PbBr_2_ was prepared in a ratio of 1.1:1 by mixing in DMSO at 45 °C in a vial and was filtered. The glass slides were cleaned by ultrasonics in ethyl alcohol and acetone and were dried. The films were coated on glass slides using spin coating and drip and dried methods. The spin coating technique could not yield a smooth film while the dripped and dried method generated a film comprising small crystals of CsPbBr_3_ including Cs_2_PbBr_4_ and PbBr_2_·2[(CH_3_)_2_SO] as shown in Figure 1b. To overcome this issue, we prepared precipitates of CsPbBr_3_ in the first step. The solution was brought to 25 °C, and acetone was added in small parts of 5 mL each time and was stirred. This process yielded the precipitates of the CsPbBr_3_. When the stirring was stopped, the precipitates of CsPbBr_3_ settled at the bottom of the vial. The solution above the precipitates was removed immediately using a pipet. The process of adding acetone and removing the solution from the top of the precipitates was repeated a few times until there was no DMSO left inside the vial. Some parts of the CsPbBr_3_ precipitates were dried at 80 °C for 3 h to perform structural analysis. To form a film, the precipitates dispersed in acetone were dripped on a glass slide and were annealed at 80 °C to get CsPbBr_3_ film. In a separate vial, a mixture of 0.5 g of poly(methyl methacrylate) (PMMA) and 5 mL of acetone was prepared at 30 °C. A small amount of PMMA solution (1 mL) was taken, added to the precipitates of CsPbBr_3_, and the resulting suspension was stirred for 1 h. Then, the solution containing PMMA and CsPbBr_3_ was dripped on a glass slide and annealed at 80 °C.

The SC of CsPbBr_3_ is synthesized using inverse temperature crystallization (ITC) described in Figure 1d.^42^ A precursor solution containing cesium bromide (CsBr) and lead bromide (PbBr_2_) in a 1:2 ratio and choline bromide was prepared in dimethyl sulfoxide (DMSO) at 45 °C in a vial and filtered. After that, the solution was placed in a silicone oil bath to maintain a uniform temperature across the solution in the vial and was heated to 80 °C at a rate of 2 °C/h. The temperature of the solution was maintained at 80 °C for a few days. A large SC of 1.3 mm × 9 mm × 5 mm was achieved as shown in Figure 1f. The solution was cooled to 45 °C at a rate of 5 °C/h. The SC was removed from the solution, washed with dimethylformamide (DMF), and dried. The SC was cut to a small size to avoid defects and was polished for further studies.

### XRD Analysis

At room temperature, powder X-ray diffraction (PXRD) measurements were carried out on a Rigaku MiniFlex 600 diffractometer, filtering Cu Kα radiation with wavelength λ = 1.5418 Å. SC of CsPbBr_3_ and precipitates were grounded separately in an agate mortar and pestle to take the PXRD pattern. Data was collected between 5 and 75° in 2θ with a step size of 0.02° at a speed of 3 s per step counting time. The PXRD pattern was matched with that reported in refs (11,43), and (44).

### SEM and EDS Analyses

The integrated EDAX Pegasus EDS & EBSD system in FEI Quanta 3D FEG FIB/SEM was used to perform SEM analysis and the chemical composition analysis of CsPbBr_3_ SC and precipitates.

### Thermogravimetric Analysis (TGA)

Thermogravimetric analysis (TGA) was conducted by using a TA Instruments Discovery TGA550 instrument with a nitrogen purge flow rate of 100 mL/min. The sample underwent heating to 600 °C at a rate of 10 °C/min. The decomposition temperature (*T*_d_) was determined by identifying the onset point of the maximum weight loss rate.

### Differential Scanning Calorimetry (DSC)

Differential scanning calorimetry (DSC) experiments were performed using a TA Instruments Discovery DSC250 instrument under a nitrogen flow rate of 50 mL/min, with T Zero Aluminum pans employed. The experimental procedure comprised the following steps: (1) equilibration at −40 °C followed by a ramping to 150 °C at a rate of 10 °C/min; (2) ramping from 10 °C down to −40 °C; (3) further ramping to 400 °C at 10 °C/min; (4) subsequent ramping down to −40 °C at 10 °C/min; (5) final ramping to −40 °C at 10 °C/min.

### Electrical Measurements

The electrical measurements were performed on a Keithley 2450 source meter using the four-wire method shown in Figure S1a. The Ag paste was used to make contacts. The experiments were performed in a dark room. The current–voltage (*I*–*V*) characteristics were taken from 0 to −20 V and current–time (*I*–time) characteristics were collected at −20 V for all experiments. A 532 nm laser of 30 mW and a 375 nm ultraviolet source of 100 mW was used. To perform the γ-radiation based electrical response, Co-57 of 20 μCi and Cs-137 of 5 μCi were used. The distance between the 532 and 375 nm lasers and the device was maintained at 20 cm while radiation sources were placed 1 cm above the device during measurements. The mobility-lifetime product (μτ) is obtained by fitting the photocurrent curve using the modified Hecht equation provided in refs (45−47). The data set used for this fitting is collected on samples with Au electrodes.
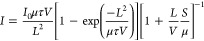
1where *I*_0_ is the saturated photocurrent, μ is the carrier mobility, τ is the carrier lifetime, *V* is the applied bias, and *L* is the thickness of the device.

## Results and Discussion

### XRD Analysis

**Figure 2:**
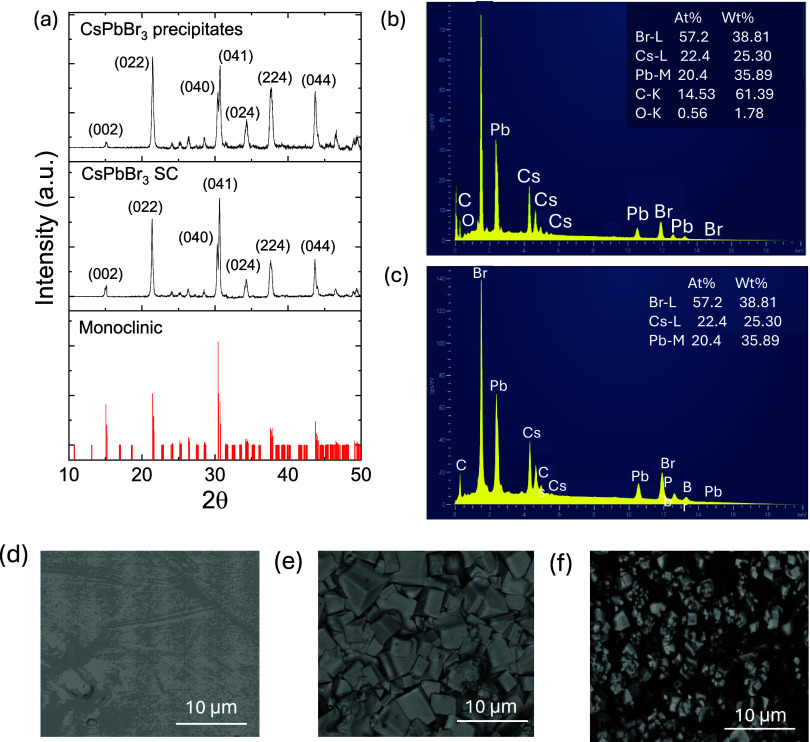
(a) PXRD pattern of CsPbBr_3_ SC and precipitates. (b) EDS map for SC and (c) precipitates of CsPbBr_3_ from EDS analysis. Panels (d)–(f) are the surface topology images for CsPbBr_3_ SC, undoped, and PMMA-doped films.

Figure 2a shows the XRD patterns of the CsPbBr_3_ SC and precipitates collected at room temperature. The peaks are comparable with the monoclinic structure of the CsPbBr_3_ at room temperature reported in refs (11,43), and (44). A slight shift in peak position toward the large 2θ is observed for the XRD pattern of CsPbBr_3_ precipitates. The lattice parameters for SC of CsPbBr_3_ are 11.727, 11.704, and 11.782 Å, and the β angle is 91.1° while it is 11.5888, 11.648, and 11.705 Å and a β angle of 92.2° for CsPbBr_3_ precipitates. The value of the the lattice parameter of the csPbBr_3_ precipitates is slightly smaller than the SC while the β angle is somewhat larger. The smaller lattice parameters and larger β angle in the CsPbBr_3_ precipitates compared to those in the SC are likely due to internal stress, defects, and possibly slight compositional variations. These structural differences have important implications for the material’s physical properties like electronic, optical, and mechanical properties, and their performance in applications like photovoltaics, LEDs, and other optoelectronic devices.

### SEM and EDS Analysis

The compositional analysis of the SC and precipitates of CsPbBr_3_ is performed by EDS analysis and is shown in Figure 2b,c. The study indicates that the ratio of Cs, Pb, and Br is approximately 1:1:3, consistent with that of CsPbBr_3_. The scanning electron microscopy (SEM) images presented in Figure 2d–f illustrate the surface morphology of CsPbBr_3_ SC, undoped, and PMMA-doped films, highlighting differences in the microstructural characteristics. The CsPbBr_3_ SC in Figure 2d exhibits a smooth and featureless surface indicative of its well-defined crystalline nature with minimal grain boundaries. In contrast, the undoped CsPbBr_3_ film (Figure 2e) reveals a polycrystalline morphology with distinct, well-faceted grains, suggesting efficient crystallization during film formation. Upon PMMA doping (Figure 2f), the microstructure becomes significantly altered, displaying a finer distribution of smaller grains embedded within the PMMA matrix.

### TGA and DSC Analysis

**Figure 3:**
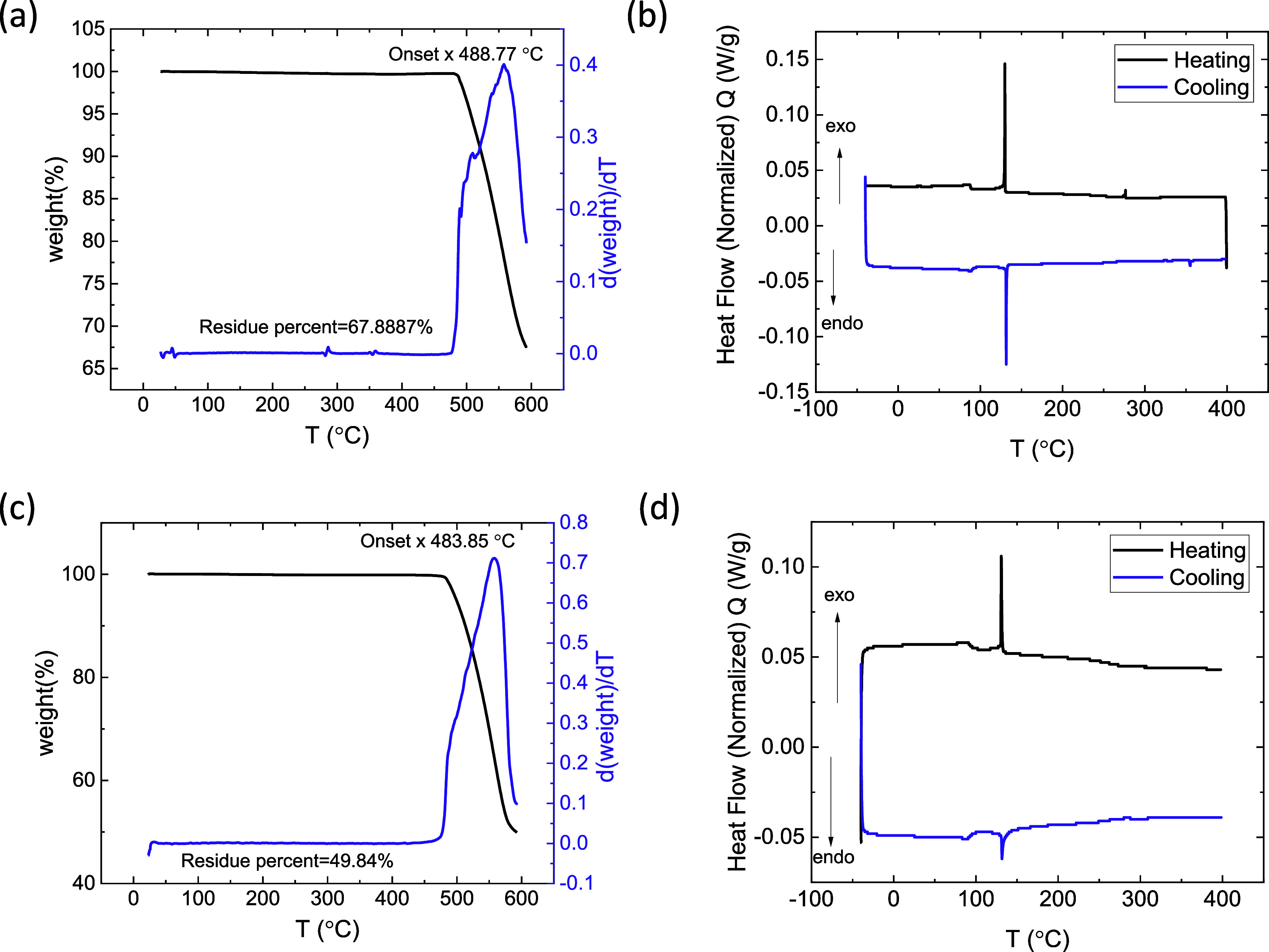
(a, b) TGA and DSC analysis of SC and (c, d) precipitates of CsPbBr_3_.

The TGA and DSC analyses of the CsPbBr_3_ SC and precipitates are shown in Figure 3a–d. CsPbBr_3_ SC prepared by the ITC method is stable up to 488.77 °C, while 33% of mass loss was seen at the melting temperature (616 °C). Whereas the precipitates are found to be stable at 483.85 °C with a mass loss of 50% at 576.85 °C. In TGA tests, a compound in precipitate form typically leaves less residue than its SC form due to several factors. Precipitates have a higher surface area, allowing for more interaction with the environment and leading to faster and more complete decomposition. Additionally, the smaller particle size of precipitates improves heat transfer and accelerates chemical reactions compared to SCs, which have a more stable and ordered atomic structure. SCs also maintain their integrity better at higher temperatures, resisting decomposition, while precipitates break down more easily, contributing to their faster decomposition and lower remaining mass. DSC analysis shows a prominent change in phase at 136 °C for both SC and precipitates of CsPbBr_3_, which is the transition temperature to cubic, consistent with other reports in the literature, with voltage.^43^

### Electrical Response of CsPbBr_3_ SC and Films

#### Current–Voltage (*I*–*V*) Characteristics

**Figure 4:**
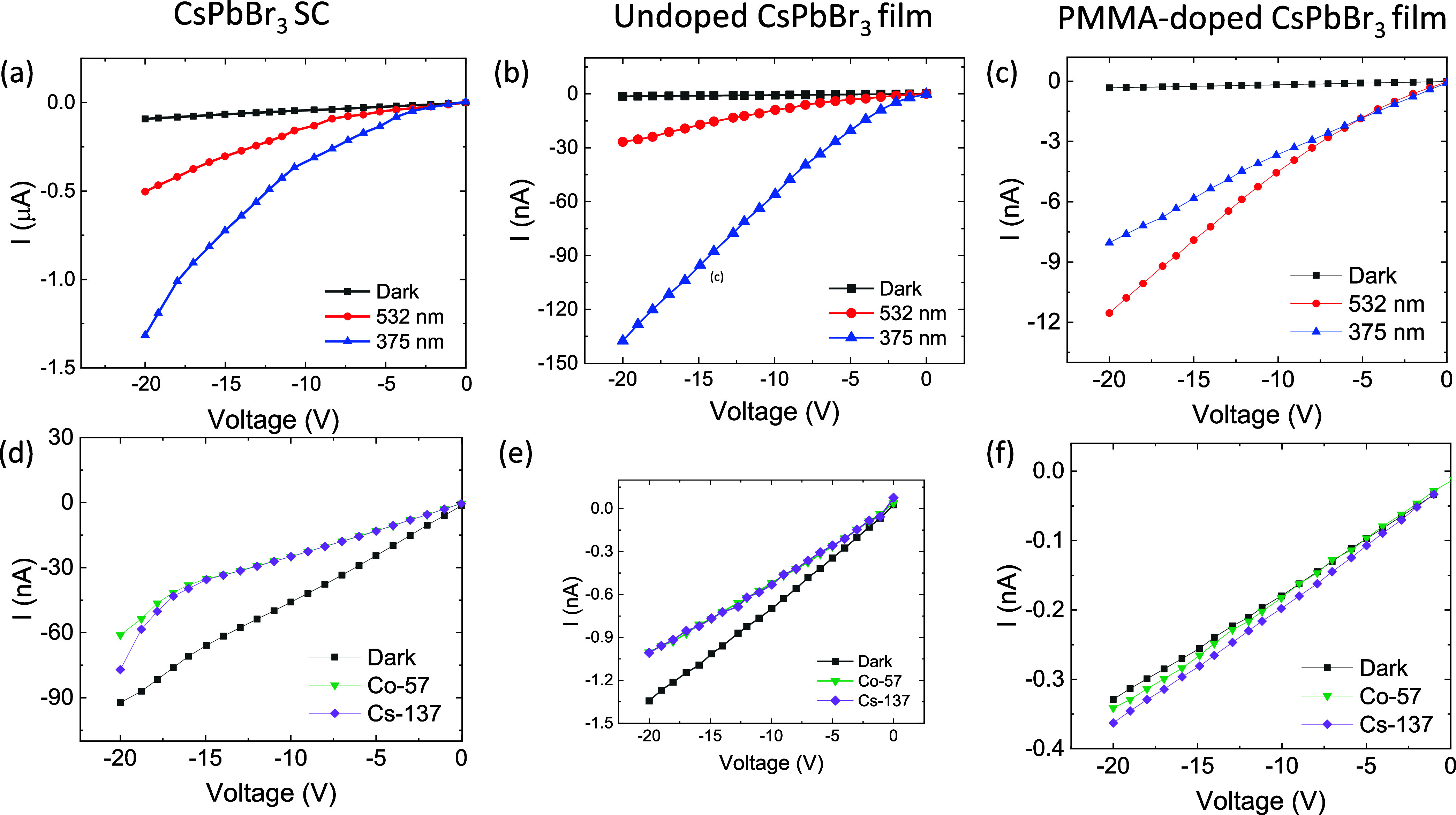
(a–c) *I*–*V* characteristic of CsPbBr_3_ SC and undoped film and PMMA-doped film under photons of 532 and 375 nm and (d–f) under γ-radiation from Co-57 and Cs-137.

The current–voltage (*I*–*V*) characteristics of the SC and undoped and PMMA-doped CsPbBr_3_ film under 532, 375 nm and γ-radiation from Co-57 and Cs-137 are shown in Figure 4, with voltage swept from 0 to −20 V. Figure 4a illustrates the *I*–*V* characteristics of an SC under three different conditions: dark, under illumination at 532 nm, and under illumination at 375 nm. In the dark, the current remains almost constant and near zero across the applied voltage range from 0 to −20 V, indicating minimal charge carrier generation without light. Under 532 nm illumination, a noticeable increase in current is observed, showing a more significant negative photocurrent as the voltage decreases, suggesting increased carrier generation due to photon absorption at this wavelength. The most substantial response occurs under 375 nm illumination, where the current reaches more negative values, indicating that this wavelength has the highest interactions with the crystal, likely due to a higher energy photon capable of generating more electron–hole pairs. The overall trend indicates that SC exhibits photoconductivity, with stronger responses at shorter wavelengths, consistent with the material’s absorption characteristics.^48^ A similar behavior is observed in the current–voltage characteristics of the undoped film of CsPbBr_3_ when exposed to 532 and 375 nm as shown in Figure 4b. The current in the undoped CsPbBr_3_ film is half of the magnitude of the SC, while the increase in the slope of the current remains comparable to that of the SC. Additionally, the current response under 375 nm illumination is four times higher than that under 532 nm, which aligns with the SC response, where the current is three times greater for 375 nm compared to that at 532 nm. However, a different behavior is observed in the *I*–*V* characteristics of the PMMA-doped CsPbBr_3_ film, as shown in Figure 4c. From 0 to −5 V, the current generated under an illumination of 375 nm is higher than that under 532 nm. However, beyond 5 V, the slope of the current under 532 nm increases sharply, and the current surpasses that of 375 nm, becoming 1.5 times larger at −20 V.

Figure 4d–f shows the *I*–*V* characteristic of CsPbBr_3_ SC, undoped film, and PMMA-doped film under dark and γ-radiation from Co-57 and Cs-137. The CsPbBr_3_ SC’s current under γ-radiation from Co-57 and Cs-137 is smaller than the dark current from 0 to – 20 V. The current from Cs-137 and Co-57 sources is linear and almost the same in magnitude from 0 to −15 V. But above −15 V, the value of the current changes significantly by increasing the slope of the current. An increase in slope indicates the charge carriers have enough energy to detrap from and contribute to the current. Figure 4e shows the *I*–*V* characteristic of the undoped film of CsPbBr_3_. Under γ-radiation, the current is smaller than the dark current. Another feature worth noticing is the slope of the *I*–*V* curve under radiation. The slope of the *I*–*V* curve is reducing under γ-radiation as compared to the dark current that maintains a steady slope. This feature suggests that the charge carriers generated by γ-radiation are being trapped by defects in the undoped film. This feature is predictable as the undoped film constitutes the interface between particles, constituting more defects than the SC. Furthermore, the current undoped film is smaller by three orders in magnitude from the SC. The *I*–*V* characteristic of PMMA-doped CsPbBr_3_ shown in Figure 4f is different from CsPbBr_3_ SC and undoped film. The current under γ-radiation is greater than the dark current. Whereas the slope of the current is reduced after −10 V. This suggests that the charge carriers generated under γ-radiation have enough energy to drift to the electrode and cause an increase in the current significantly. Meanwhile, the γ-radiation generates defects in the film that trap the charge carriers, hence reducing the slope after −10 V.

**Figure 5:**
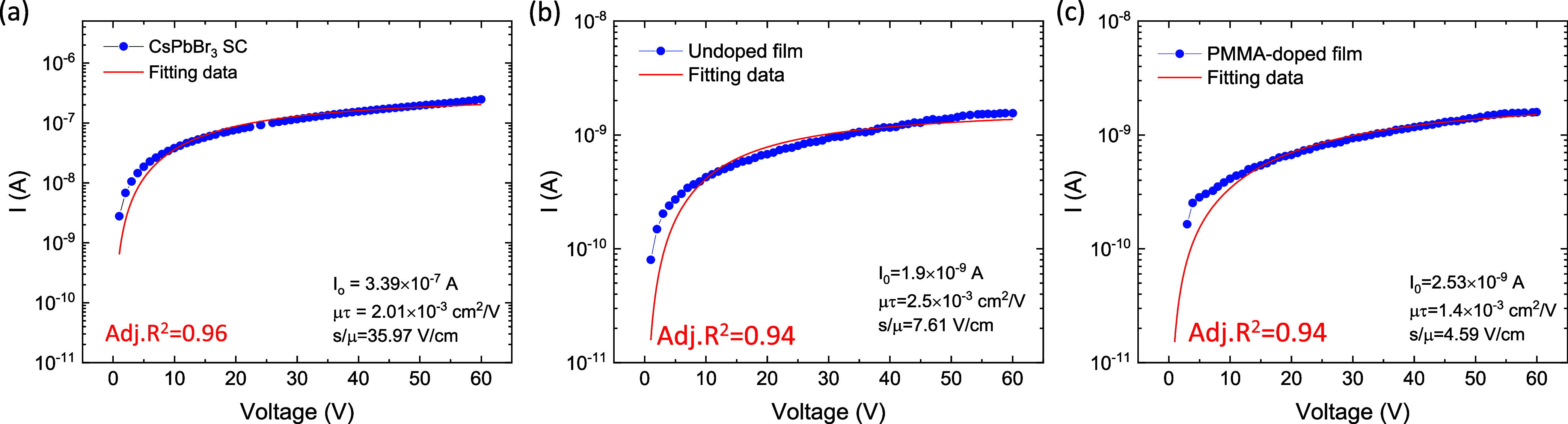
(a–c) Modified Hecht equation fitting for SC, undoped, and PMMA-doped CsPbBr_3_ films performed on data collected with Au electrodes on all three devices.

The fitting of the photocurrent curves using the modified Hecht equation is shown in Figure 5a–c. The fitting highlights the variations in carrier transport and surface recombination characteristics across a CsPbBr_3_ single crystal (SC), undoped CsPbBr_3_ film, and PMMA-doped CsPbBr_3_ film. For the SC device (Figure 5a), the highest mobility-lifetime product (μτ) value indicates superior carrier transport efficiency and collection due to its crystalline nature, resulting in minimal scattering and recombination losses. The undoped film (Figure 5b) shows a slightly lower μτ value compared to the SC, consistent with the presence of grain boundaries introducing additional scattering while maintaining a reasonable charge transport efficiency. In contrast, the PMMA-doped film (Figure 5c) exhibits the lowest μτ value, reflecting the influence of PMMA passivation, which reduces the trap density but introduces insulating effects, thereby limiting charge transport. The surface recombination velocity (*S*) and adjusted *R*^2^ values across all samples confirm the quality of the fits, emphasizing that SC provides the best charge transport performance.

#### Current–Time (*I*–*t*) Characteristics

**Figure 6:**
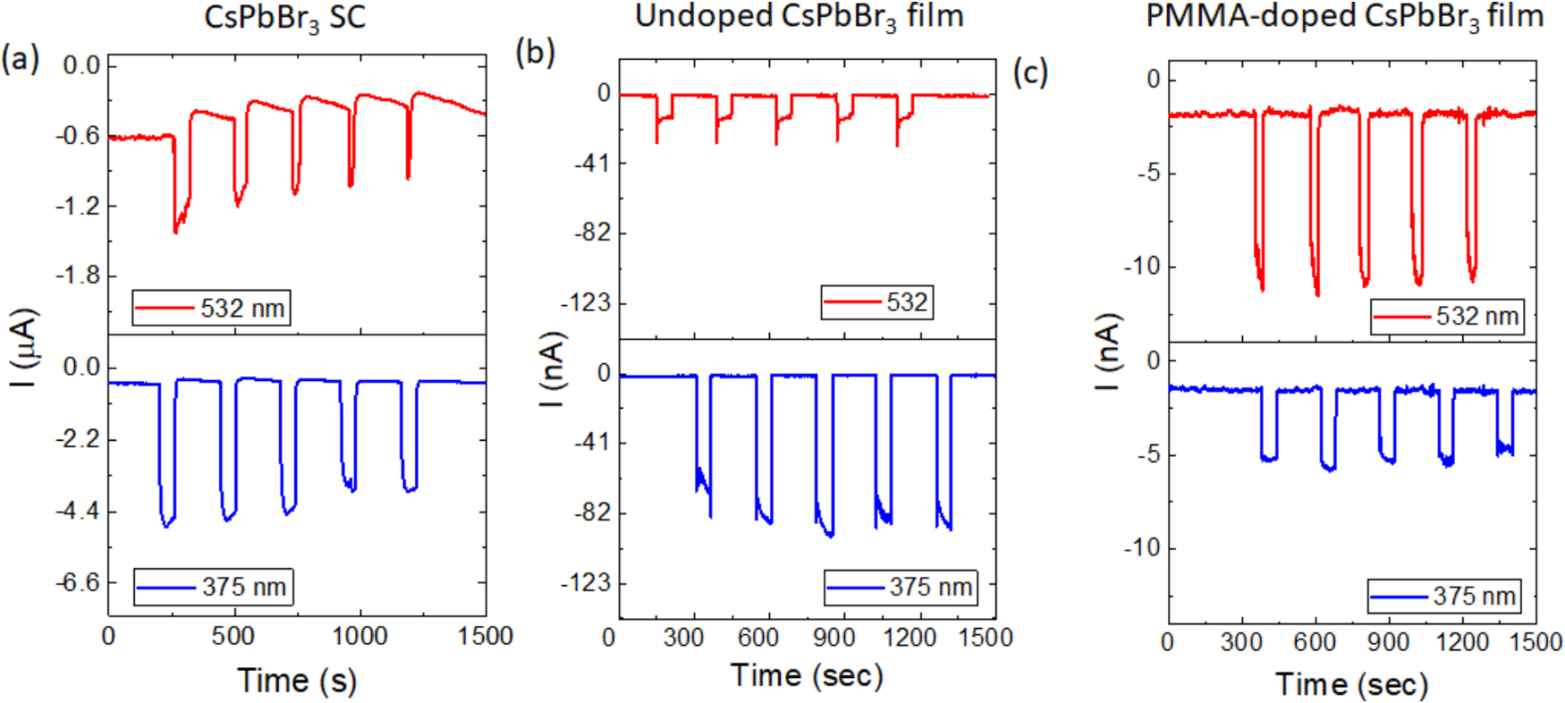
*I*–*t* characteristics collected at −20 V for (a) CsPbBr_3_ SC, (b) undoped film, and (c) PMMA-doped film.

The current–time (*I*–*t*) characteristics of the CsPbBr_3_ SC, undoped film, and PMMA-doped film, measured at −20 V, are shown in Figures 6a–c and S2a. For the CsPbBr_3_ SC under 532 nm illumination, the first exposure produces the highest current response of −1.4 μA. However, during the second exposure, the current decreases to −1.3 μA. In the subsequent three exposures, the response stabilizes around −1.1 μA. A decrease in dark current after each exposure from its precedent dark current value is observed. In contrast, the response under 375 nm illumination exhibits the opposite trend. The first exposure results in a minimal current of −5.3 μA, but with each successive exposure, the current either remains the same or decreases. In comparison, the *I*–*t* characteristics of the undoped CsPbBr_3_ film show much lower current responses under both 532 and 375 nm illumination relative to the SC. Notably, the response to 375 nm is four times larger than to 532 nm in the undoped film. The needle-like rise in current under 532 nm has been reported previously.^49^ The PMMA-doped CsPbBr_3_ film displays an opposite trend compared to the SC and undoped film. The PMMA-doped film exhibits a stronger response to 532 nm illumination than to 375 nm, suggesting that the electronic states in the PMMA-doped CsPbBr_3_ film are more compatible with 532 nm excitation.

**Figure 7:**
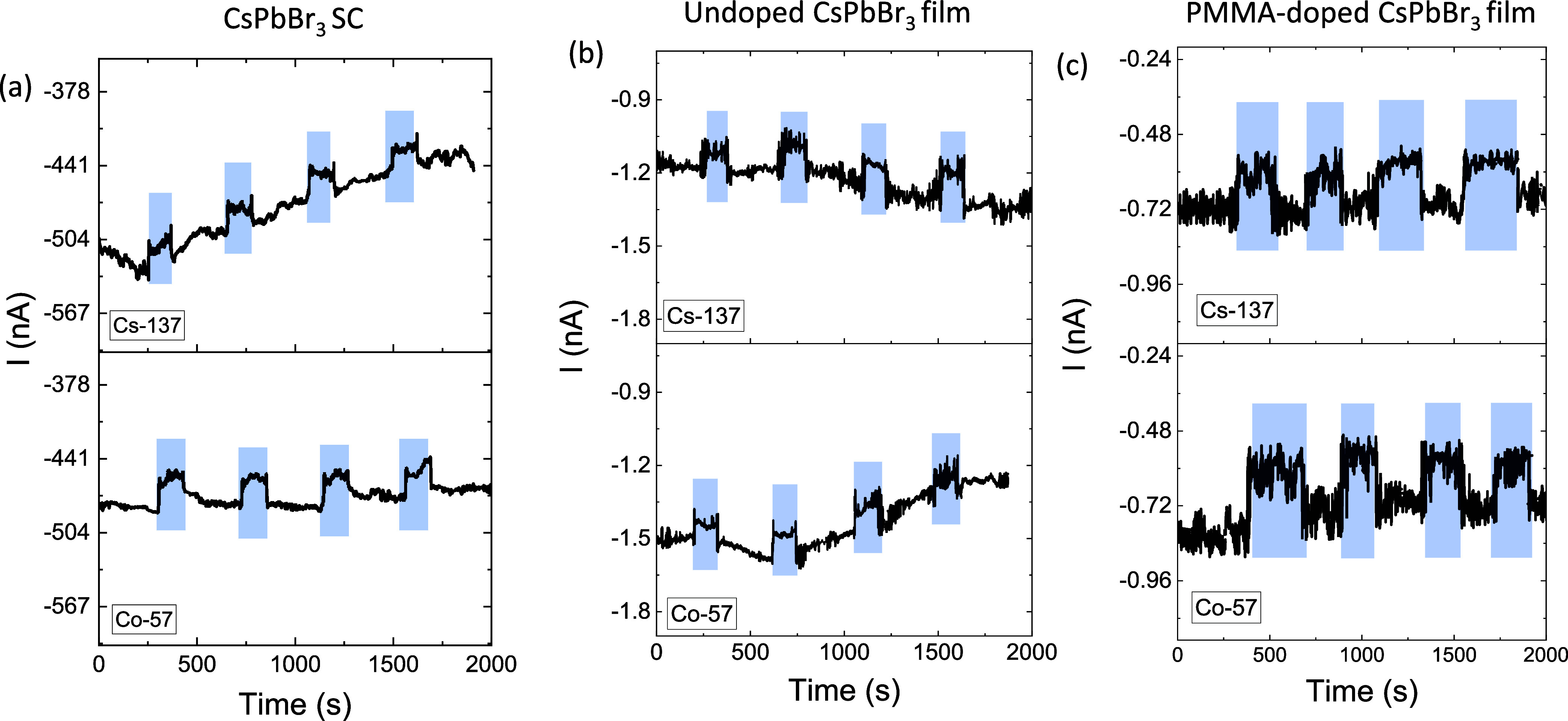
*I*–*t* characteristics under γ-radiation from Co-57 and Cs-137 taken at −20 V for (a) CsPbBr_3_ SC, (b) undoped film, and (c) PMMA-doped CsPbBr_3_ film. The blue regions correspond to the exposure to γ radiation from Cs-137 and Co-57.

Figures 7 and S2b present the time-based electrical response of SC, undoped film, and PMMA-doped CsPbBr_3_ film—when they were exposed to γ-radiation from Cs-137 and Co-57 sources (expressed with blue regions) at an applied bias of −20 V. For the SC and undoped film of CsPbBr_3_, the dark current is not stable under dark conditions. This unstable behavior in the absence of radiation indicates an inherent instability in the SC and undoped film, possibly due to^32^ humidity effects and intrinsic defects or charge-trapping mechanisms that cause current fluctuations even without γ-radiation.^32,50^ While under γ-radiations, a distinct change in current is observed for both SC and undoped film of CsPbBr_3_. The sharp peaks followed by gradual recovery suggest that the SC and undoped film undergo transient electrical changes due to radiation exposure. This behavior is likely linked to charge trapping and recombination events within the material. The heightened sensitivity to Cs-137 may arise from interactions between γ-photons and defects or traps present in the film. The PMMA-doped film also demonstrates a decrease in current under γ-radiation, contrary to the *I*–*V* characteristics. A possible factor is that the charge carriers are generated before applying bias voltage while tracing the *I*–*V* characteristic that can lead to an increase in the current while the charge carriers are produced under γ-radiation exposure that is already biased; these increased charge carriers can contribute to an increase in scattering centers and hence reducing the current under γ-radiation. The substantial noise and broad fluctuations in current could be associated with the polymer matrix’s reaction to γ-irradiation.

The differences in behavior among CsPbBr_3_ SC, undoped, and PMMA-doped films arise from variations in the material structure, defect states, and the role of PMMA as a polymer matrix. SC, with its defect-free crystalline nature, exhibits superior charge transport and minimal recombination, as indicated by its high mobility-lifetime product (μτ) in Hecht fitting, consistent with efficient carrier collection and low scattering losses. Its *I*–*V* characteristics reveal strong photoconductivity, particularly under 375 nm illumination, where higher photon energy generates more electron–hole pairs. In contrast, the undoped film demonstrates reduced performance due to the presence of grain boundaries and intrinsic defects that act as charge traps, limiting mobility and conductivity. Its lower μτ value compared to that of SC reflects increased recombination and scattering, while its weaker current response to illumination and γ-radiation highlights the impact of structural imperfections. The PMMA-doped film introduces a different dynamic, as the polymer matrix enhances uniformity and reduces defect states, initially improving charge transport under lower voltages. However, the insulating nature of PMMA increases resistance and introduces dielectric barriers, leading to reduced overall mobility and a lower μτ value compared to both SC and undoped films. The PMMA matrix also modifies the optical bandgap, resulting in a steeper current rise under 532 nm illumination at higher voltages, surpassing the 375 nm response.^27−29^ Under γ-radiation, the SC shows linear current changes due to efficient detrapping of carriers at higher biases, while the undoped film exhibits significant current reduction due to enhanced trapping at defects. The PMMA-doped film, though showing currents higher than those of the undoped film under γ-radiation, suffers from increased scattering and polarization effects introduced by the polymer. These findings highlight the role of material structure, from the crystalline integrity of SC to the defect-prone nature of undoped films and the insulating yet stabilizing effects of PMMA, in shaping the electrical and optical responses of CsPbBr_3_-based devices.

## Conclusions

In this study, we have systematically explored the electrical behavior of CsPbBr_3_ single crystals, as well as undoped and PMMA-doped films, under γ-radiation. A novel growth method for both undoped and doped films was introduced, resulting in high-quality materials with XRD and EDS analyses confirming their purity and crystallographic integrity comparable to single crystals. The current–voltage measurements revealed that single crystal and undoped CsPbBr_3_ films exhibit enhanced sensitivity to 375 nm light, while the PMMA-doped film showed a heightened response to 532 nm wavelengths. Furthermore, the behavior under γ-radiation exhibited distinct trends. For single crystals and undoped films, the current under γ-radiation was consistently lower than the dark current, while the PMMA-doped film displayed an increase in current under the same conditions. When analyzing the current–time characteristics at −20 V, the current under γ-radiation was less negative than the dark current. The mobility-lifetime product (μτ) is highest for the CsPbBr_3_ single crystal (SC), moderate for the undoped film, and lowest for the PMMA-doped film, reflecting a trade-off between carrier transport efficiency and defect passivation. These observations contribute to a deeper understanding of the material’s behavior under visible and high-energy photons, providing valuable insights for designing optoelectronic devices and high-energy radiation detectors. Future research can be built on these findings to optimize CsPbBr_3_-based devices for targeted applications in advanced sensing technologies.

## Supporting Information Available

Schematic diagram of devices for electrical measurement, dark current for CsPbBr_3_ SC as a function of time with Ag-contacts, *I*–*t* and *I*–*V* characteristics of CsPbBr_3_ SC with Au contacts under 532, 375 nm and γ-radiation, *I*–*V* characteristics of CsPbBr_3_ Sc, undoped and PMMA-doped films with Ag-contacts under γ-radiation from 0 to −60 V, γ-radiation spectrum of commercially available CZT detectors and CsPbBr_3_ single crystal (PDF)
