# Supplementary material for: Study of the Electrical Behavior of CsPbBr3 Single Crystal and Films under Visible and High-Energy Photons
Source: ACS Appl Opt Mater. 2025 Mar 20;3(3):620–9. doi: 10.1021/acsaom.4c00455 (PMC11959612; doi:10.1021/acsaom.4c00455)
Supplement: Supplementary file 1 — ot4c00455_si_001.pdf [file ot4c00455_si_001.pdf]

Supporting Information

The study of the electrical behavior of CsPbBr3 single crystal and films under visible and high-energy photons

Tahira Khan[†, *], Manas R. Gartia[§, *] Jianwei Wang[⊥, #,] and Jyotsna Sharma[†]

[†] Department of Petroleum Engineering, Louisiana State University, Baton Rouge, Louisiana 70803, United States
[§] Department of Mechanical and Industrial Engineering, Louisiana State University, Baton Rouge, Louisiana 70803, United States
[⊥] Department of Geology and Geophysics, Louisiana State University, Baton Rouge, Louisiana 70803, United States
[#] Center for Computation and Technology, Louisiana State University, Baton Rouge, Louisiana 70803, United States

*Corresponding author; email: mgartia@lsu.edu, tkhan@lsu.edu

Figure S1 (a) a schematic diagram of the I-V and I-t test. (b) Dark current for $CsPbBr_3$ SC over time with Ag contacts. 3

Figure S2 The I-t characteristics of $CsPbBr_3$ Sc with Au contacts under (a) 532 and 375 nm and (b) γ-radiation. 4

Figure S3 The I-V characteristics of $CsPbBr_3$ Sc with Au contacts under (a) 532 and 375 nm and (b) γ-radiation. (c) The I-V characteristics of $CsPbBr_3$ Sc with Au contacts under γ-radiation from 0 to –60 V. 5

Figure S4 The I-V characteristics of $CsPbBr_3$ Sc, undoped and PMMA-doped films with Ag contacts under γ-radiation from 0 to –60 V. 6

Fig. S5 (a) The γ-radiation spectrum of commercially available CZT detectors and for (b) $CsPbBr_3$ SC (synthesized at 80 °C) under Co-57 at +500 V. 7

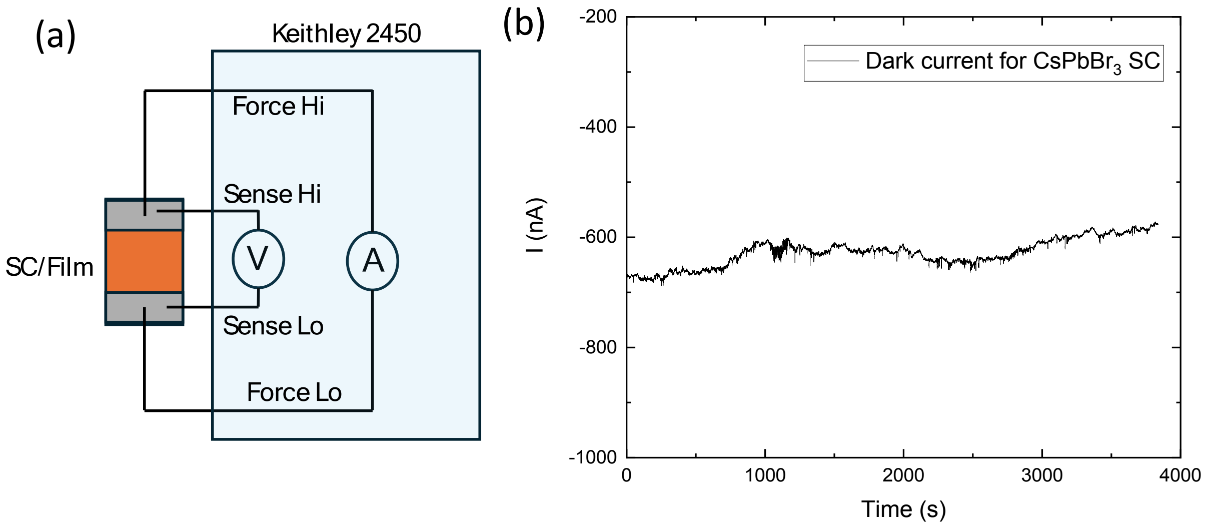

Figure S1 (a) a schematic diagram of the I-V and I-t test. (b) Dark current for $CsPbBr_3$ SC over time with Ag contacts.

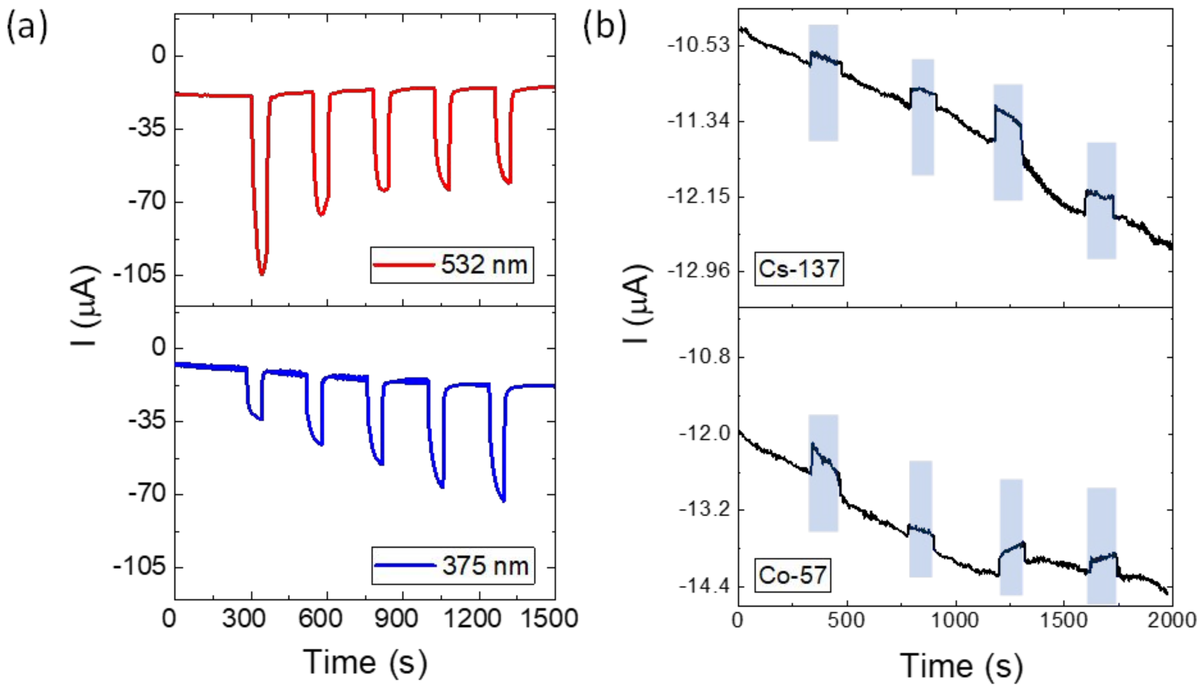

Figure S2 The I-t characteristics of $CsPbBr_3$ Sc with Au contacts under (a) 532 and 375 nm and (b) γ-radiation.

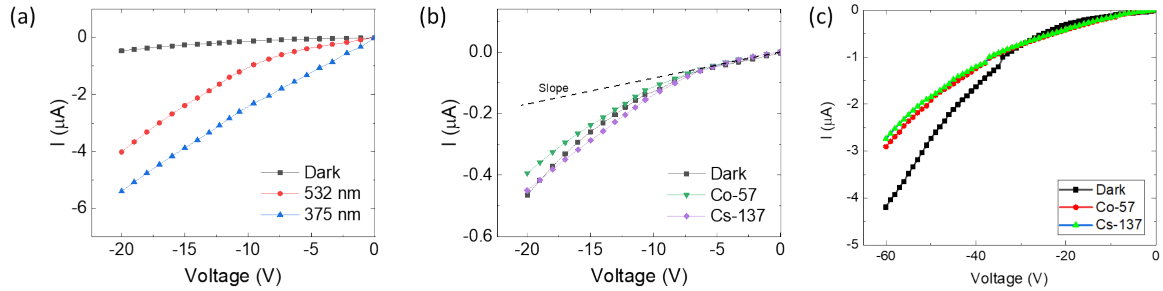

Figure S3 The I-V characteristics of $CsPbBr_3$ Sc with Au contacts under (a) 532 and 375 nm and (b) γ-radiation. (c) The I-V characteristics of $CsPbBr_3$ SC with Ag contacts under γ-radiation from 0 to –60 V.

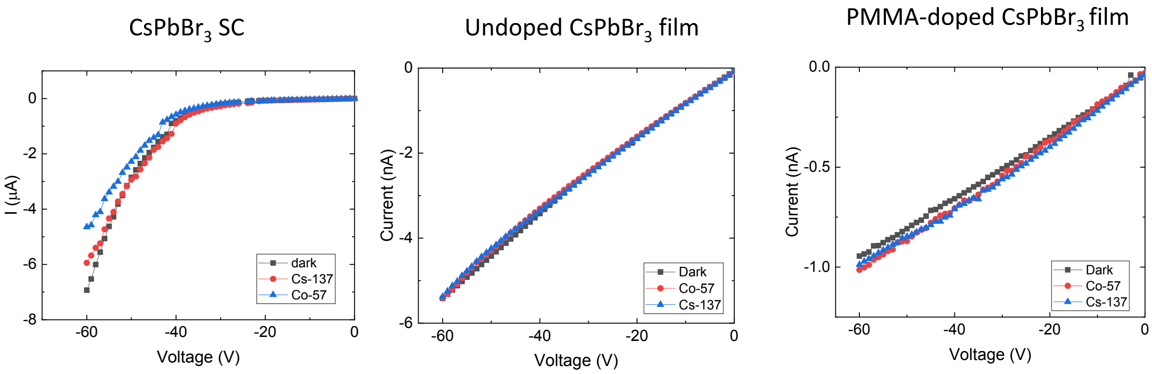

Figure S4 The I-V characteristics of $CsPbBr_3$ Sc, undoped and PMMA-doped films with Ag contacts under γ-radiation from 0 to –60 V.

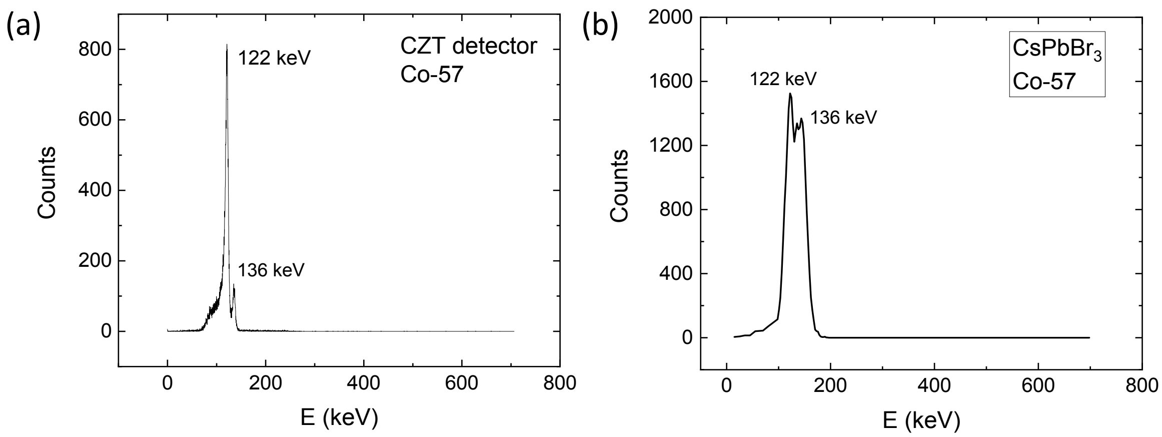

Fig. S5 (a) The γ-radiation spectrum of commercially available CZT detectors and for (b) $CsPbBr_3$ SC (synthesized at 80 °C) under Co-57 at +500 V.
